# Current-induced crystallisation in Heusler alloy films for memory potentiation in neuromorphic computation

**DOI:** 10.1038/s41598-021-96706-9

**Published:** 2021-08-30

**Authors:** William Frost, Kelvin Elphick, Marjan Samiepour, Atsufumi Hirohata

**Affiliations:** 1 Department of Physics, University of York, York, YO10 5DD UK; 2 Department of Electronic Engineering, University of York, York, YO10 5DD UK; 3 Present Address: Oxford Instruments Plasma Technology, Bristol, BS49 4AP UK; 4 Present Address: Taiwan Semiconductor Manufacturing Company, Hsinchu, 300-78 Taiwan, R.O.C.; 5 Present Address: Seagate Technology, Londonderry, BT48 0LY UK

**Keywords:** Spintronics, Magnetic properties and materials

## Abstract

The current information technology has been developed based on von Neumann type computation. In order to sustain the rate of development, it is essential to investigate alternative technologies. In a next-generation computation, an important feature is memory potentiation, which has been overlooked to date. In this study, potentiation functionality is demonstrated in a giant magnetoresistive (GMR) junction consisting of a half-metallic Heusler alloy which can be a candidate of an artificial synapse while still achieving a low resistance-area product for low power consumption. Here the Heusler alloy films are grown on a (110) surface to promote layer-by-layer growth to reduce their crystallisation energy, which is comparable with Joule heating induced by a controlled current introduction. The current-induced crystallisation leads to the reduction in the corresponding resistivity, which acts as memory potentiation for an artificial GMR synaptic junction.

## Introduction

Nanoelectronic devices, namely silicon chips, have been following Moore’s law by increasing the number of transistors on a chip over the last decades. However, most recent workloads run on a chip cannot use the entirety of the transistors and their clusters, i.e., cores, causing a problem called “dark silicon”. In order to solve such inefficiency, beyond von Neumann type chip architecture has been sought.

In spintronics, a magnetic moment and an electron spin can be used as an information carrier while achieving low energy consumption^1,2^. Such a concept is to utilise resistance changes in a voltage-induced magnetisation reversal process and to control them by taking a “minor” loop in the process. Here, the data is stored as a magnetisation direction in one of the ferromagnetic layers in MTJ, which can be reversed by applying a current. However, such a voltage-tuneable system requires another device to record the input patterns. Hence, it is important to implement the data-logging functionality into the computing device.

We have recently found a process to lower the crystallisation temperature of a ternary/quaternary Heusler alloy film by growing on the (110) plane^3^. Our latest study demonstrates that almost 85% of a Co_2_FeAl_0.5_Si_0.5_ (CFAS) Heusler-alloy film can be crystallised at 353 K for 2 min^4,5^. In this study, we have demonstrated the crystallisation of a Heusler alloy film with the (110) surface in a giant magnetoresistive (GMR) junction by introducing a current pulse, of which amplitude is greater than the typical sensing current. This is a new concept and can be used as a registrar and data-logger in nanoelectroncs and neuromorphic computation.

## Results

**Figure 1:**
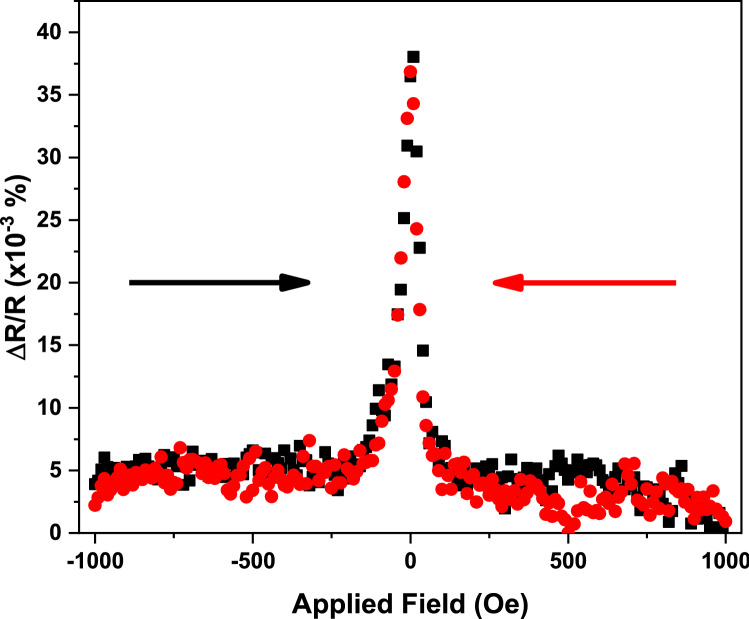
Magnetic properties of a CFAS/Ag/CFAS GMR device with the diameter of 150 nm × 100 nm. GMR curves measured under an applied field of ± 1 kOe and a sensing current of 50 µA before the current crystallisation by an applied current of 500 µA for 1 s.

Figure 1 shows a magnetoresistance curve for the GMR pillar before the current annealing. The initial GMR effect is extremely small at only 0.04% and is very unstable, with only anti-parallel configurations to be stable without magnetic field applications. This is most likely due to the lack of crystallisation in the Heusler alloy layer, which is known to form an amorphous phase in the as deposited state^3^. Such an amorphous phase reduces the magnetisation significantly and thus the GMR effect. Furthermore, possible edge damage effects caused by the ion-milling of the GMR pillar affect device properties. The interfacial roughness induced during the deposition also creates nucleation sites for the magnetisation reversal, hence a reduced coercivity resulting in a lack of stability for an antiparallel configuration. After the introduction of a current of 500 μA in a series, the shape of the GMR curves measured by a sensing current of 50 µA is maintained but the resistance values are changed as shown in the supplemental information.

**Figure 2:**
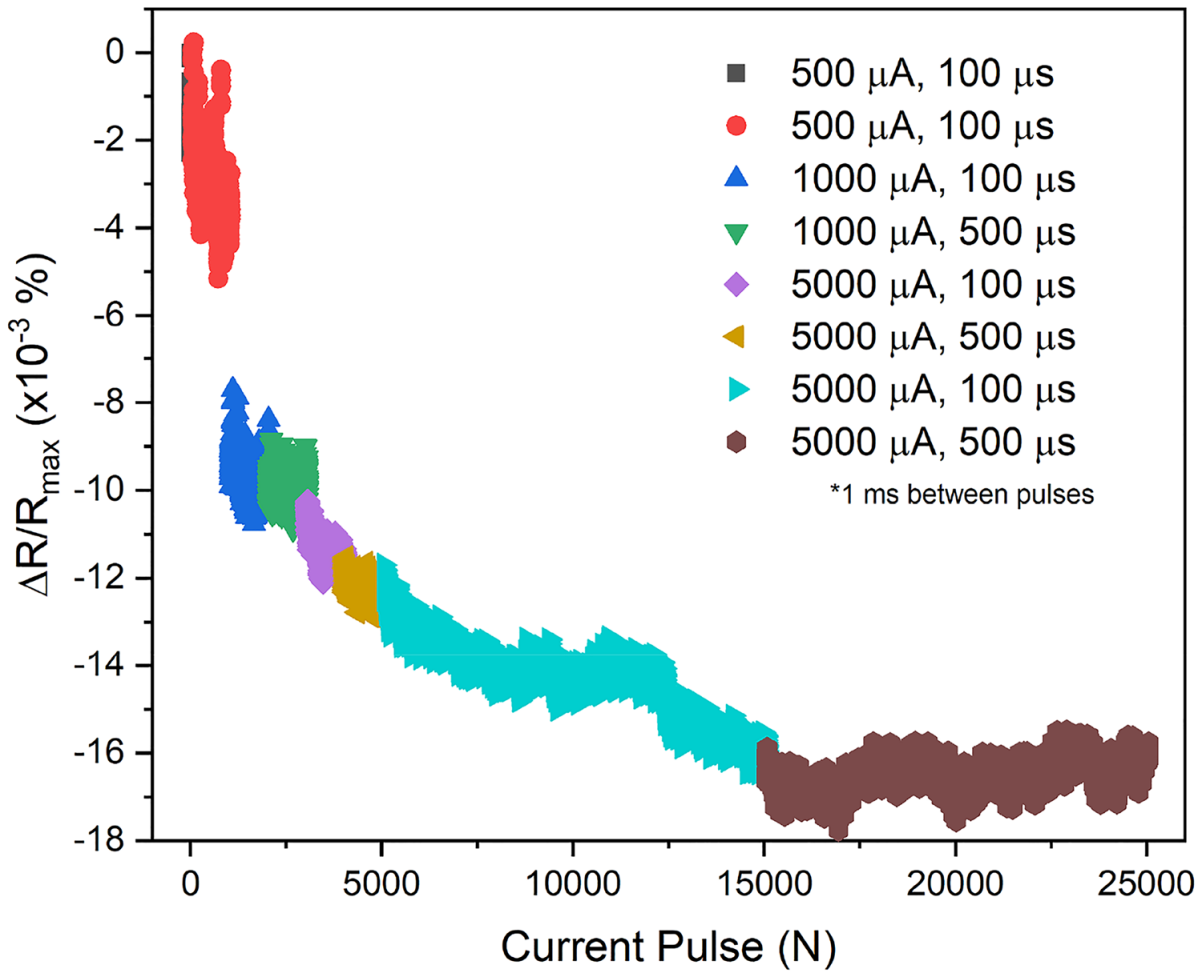
Current-induced crystallisation. Resistance change after a series of pulse current applications of 500 µA up to 5 mA for 100 µs up to 500 µs in a GMR device of CFAS/Ag/CFAS with the same diameter of 150 nm × 100 nm as that measured in Fig. 1. The GMR ratios were measured using the conventional four probe setup with flowing a sensing current of 50 µA.

The resistance changes after a series of current pulse applications of 500 µA up to 5 mA for 100 µs up to 500 µs are shown in Fig. 2 in a GMR junction, consisting of CFAS/Ag/CFAS. Note that the standard error is taken in the last 25 point of the data, resulting in a value of 200 µΩ, which is far below the changes in the resistance. By applying a pulse current up to 25,000 times with different conditions, the resistance changes almost monotonically, which is expected to be due to the competition of the CFAS Heusler-alloy crystallisation. Little change is observed at 500 μA, as in the GMR measurement in Fig. 1. After a series of current applications (current pulse *N* > 15,000 times in Fig. 2), the resistance become saturated, suggesting the current-induced crystallisation is completed. These results confirm that the GMR nanopillar can be used for neuromorphic operation as reported for a TMR nanopillar^1,2^.

Using Ohm’s law, the application of a 100 µA current for 10 s introduces 6.24 × 10^–14^ J to a Heusler alloy nanopillar (10 nm thick and 100 nm diameter), assuming the resistivity is similar to that of Co (6.24 nΩ⋅m). For an ideal case, this increases the temperature of the Heusler layer by 51.5 K, since the heat capacity of Co is 24.81 J/mol K and the density of Co is 8.90 g/cm^3^. Hence, current-induced annealing is achieved. This can offer the data-logging functionality into a neuromorphic computing device with a GMR junction. Here, the steps of the data-logging can be controlled by the amplitude and duration of the current flow.

**Figure 3:**
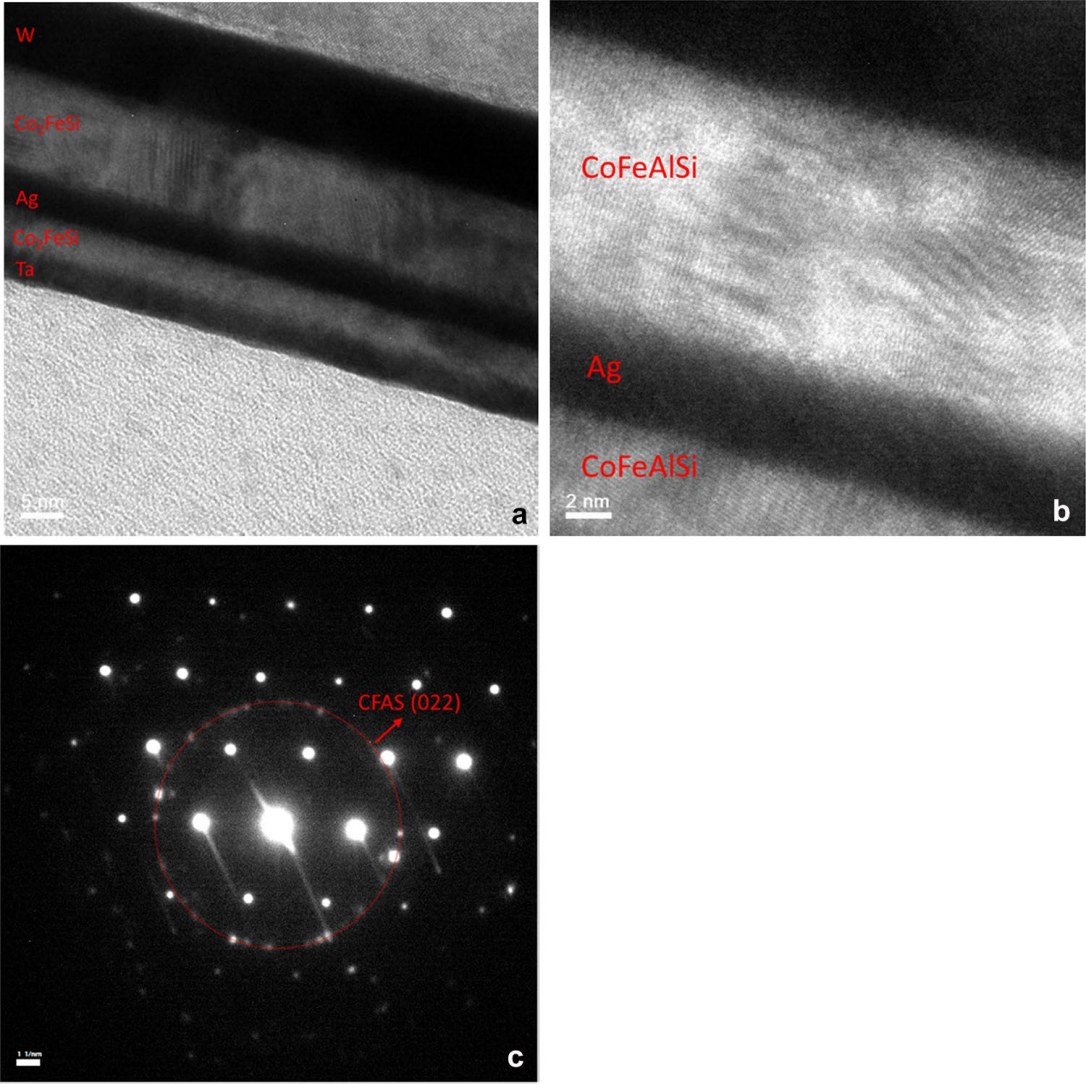
Structural analysis of crystallised GMR device by current introduction. Cross-sectional TEM images of the GMR device after the current-induced crystallisation is completed as similar to *N* > 15,000 in Fig. 2 with (**a**) 300 k and (**b**) 800 k magnification. (**c**) Diffraction pattern obtained using nanobeam.

The nanostructure and layer thicknesses of the GMR device after a series of current-induced annealing applications to saturate the resistance were investigated using cross-sectional transmission electron microscope (TEM) observation. Figure 3a,b shows bright field cross-sectional TEM images of the device with 300 k and 800 k magnification, respectively. The actual layer thicknesses of the GMR device were measured to be Si/SiO_2_//W (11.1)/CFAS (11.5)/Ag (4.2)/CFAS (4.6)/Ru (3.5) (thickness in nm). Lattice fringes of the CFAS was observed in the cross-sectional TEM image in Fig. 3b. The crystallinity of the CFAS has been confirmed using nanobeam as shown in Fig. 3c. A diffraction ring patter was observed, however some diffraction spots were also observed within the ring region. It represented CFAS was partially crystallised after current annealing took place. The CFAS(220) diffraction ring was observed at 5.1 nm^−1^ from the centre spot. Therefore, the lattice constant of CFAS is estimated to be 0.57 nm, which is 101.3% of the CFAS film grown at 673 K estimated by the corresponding XRD result previously^5^. The structural analysis via TEM imaging confirmed CFAS pillars after the current introduction was partially crystallised and maintained a smooth interface of < 1 nm roughness in the GMR junction.

The reduction in the resistance in Fig. 2 and the corresponding TEM images in Fig. 3 confirm the current-induced crystallisation into the *B*2 phase of the Heusler alloy film. By assuming the film is in a circular disc shape with the diameter of 100 nm and the thickness of 10 nm, the heating value required to increase the temperature from 300 to 353 K is calculated by multiplying the mass, specific heat capacity and increased temperature, resulting in 3.31 × 10^–11^ J using the parameters for Co for simple estimation. This value is almost comparable with the Joule heating by applying an electrical current of 1 µA into the above circular disc at a voltage of 10 µV for 1 s, i.e., 1 × 10^–11^ J. One can therefore conclude that the ternary/quaternary Heusler alloy films can be crystallised by 10 steps by simply flowing a pulsed current of 100 ms or less into the films, offering a new nanoscale device fabrication method of current-induced crystallisation.

In summary, the concept of the current-induced crystallisation has been successfully demonstrated in a Heusler-alloy GMR junction. Due to the nature of a simple electrical current introduction, a nanoelectronics device does not require annealing processes but stores the operation cycle permanently, which minimises any atomic diffusion and interfacial mixing to degrade their performance. Hence, such current-induced crystallisation is expected to be used in a variety of next-generation nanoelectronics devices, which can revolutionalise solid state memory.

## Methods

### Device fabrication

A multilayer of W (20)/Co_2_FeAl_0.5_Si_0.5_ (CFAS) Heusler-alloy (10)/W (3)/CFAS (5)/Ru (3) (thickness in nm) as optimised previously^5^ was grown on a thermally oxidised Si substrate using a high target utilisation sputtering system (PlasmaQuest, HiTUS) at room temperature. The multilayer was patterned into a nanopillar junction using a combination of electron-beam lithography and Ar-ion milling using the same process as previously reported^4,5^. Here, a seed layer, Cr/W, was patterned into a bottom electrode with a width of 100–200 nm. Just above the bottom Heusler-alloy layer was patterned into a nanopillar with a diameter between 80 and 200 nm. It should be noted that the typical fluctuation in the diameter of the nanopillar fabricated was < 10 nm, leading to a volume fluctuation of ± 4 ~ ± 27% for a 10 nm thick film. The minimisation of such fluctuation across the nanopillars is critical for their consistent operation. The nanofabrication process was carefully optimised by using a double-resist process with methyl methacrylate (MMA) and poly-methyl methacrylate (PMMA). A top electrode, Ag/Ta, was then be patterned on the nanopillar in a similar manner.

### Device characterisation

The fabricated nanopillars were measured using a probe station (HiSOL, non-magnetic probe station, HMP-400 SMS) with a conventional direct-current (dc) four-terminal method with a sensing current of 50 µA. A separate dc current between 1 µA and 10 mA for 100 µs up to 1 s was applied to the nanopillar with a constant current source (Keithley, 2400) to induce Joule heating for alloy crystallisation, while the voltage across the nanopillar was measured by a nanovoltmeter (Keithley, 2182A). The Heusler-alloy crystallisation was then observed using transmission electron microscope (TEM; JEOL, JEM-2200FS) by thinning with focused ion beam (FIB, FEI, Nova 200 Dual Beam).

## Supplementary Information

Supplementary Figures.

## Data Availability

The data that support the findings of this study are available from the corresponding author upon request.

## References

[1] Grollier J, Querlioz D, Stiles MD (2016). Spintronic nanodevices for bioinspired computing. Proc. IEEE.

[2] Manipatruni S, Nikonov DE, Young IA (2018). Beyond CMOS computing with spin and polarization. Nat. Phys..

[3] Sagar J, Fleet LR, Walsh M, Lari L, Boyes ED, Whear O, Huminiuc T, Vick A, Hirohata A (2014). Over 50% reduction in the formation energy of Co-based Heusler alloy films by two-dimensional crystallisation. Appl. Phys. Lett..

[4] Frost W, Hirohata A (2018). Heusler alloys with bcc tungsten seed layers for GMR junctions. J. Magn. Magn. Mater..

[5] Frost W, Samiepour M, Hirohata A (2019). Low-temperature deposition of Heusler alloys with perpendicular magnetic anisotropy. J. Magn. Magn. Mater..

