# Supplementary material for: Current-induced crystallisation in Heusler alloy films for memory potentiation in neuromorphic computation
Source: Sci Rep. 2021 Aug 30;11:17382. doi: 10.1038/s41598-021-96706-9 (PMC8405805; doi:10.1038/s41598-021-96706-9)
Supplement: Supplementary file 1 — Supplementary Figures. [file 41598_2021_96706_MOESM1_ESM.docx]

**Current-Induced Crystallisation in Heusler Alloy Films**

**for Memory Potentiation in Neuromorphic Computation**

William Frost,^1), a)^ Kelvin Elphick,^2), b)^ Marjan Samiepour ^2), c)^ and Atsufumi Hirohata ^2), *^

^1)^ *Department of Physics, University of York, York YO10 5DD, United Kingdom*

^2)^ *Department of Electronic Engineering, University of York, York YO10 5DD, United Kingdom*

^a)^ *Present Address: Oxford Instruments Plasma Technology, Bristol BS49 4AP, United Kingdom*

^b)^ *Present Address: Taiwan Semiconductor Manufacturing Company, Hsinchu 300-78, Taiwan, R.O.C.*

^c)^ *Present Address: Seagate Technology, Londonderry BT48 0LY, United Kingdom*

^*^ *Corresponding author: E-mail: atsufumi.hirohata@york.ac.uk*

Figure S1 shows the development of giant magnetoresistive (GMR) behaviour by an applied current of 500 µA for 1 s up to 45 times. The noise decreases with increasing the current application, proving the Co_2_FeAl_0.5_Si_0.5_ (CFAS) Heusler-alloy films in the GMR pillar device. Similar behaviour has been observed in over 12 devices.

(a)
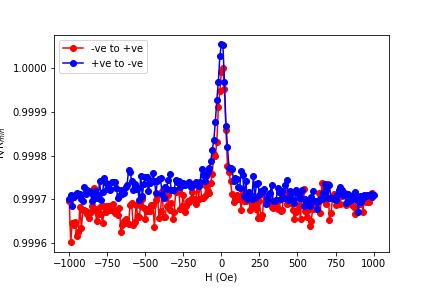
 (b)
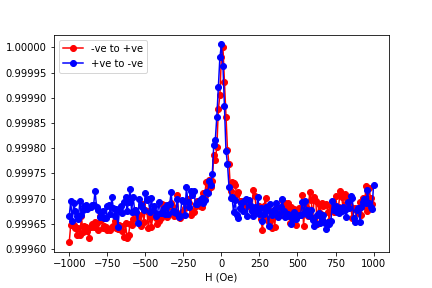


***R*/*R*_min_ (%)**

(c)
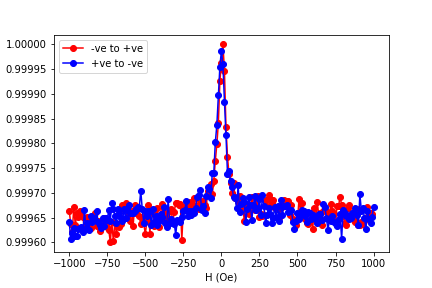
 (d)
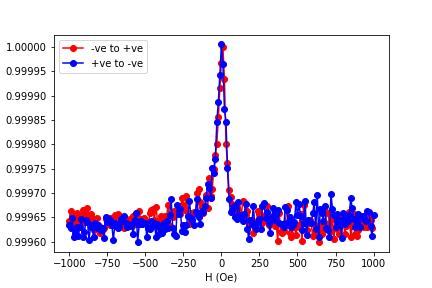


(e)
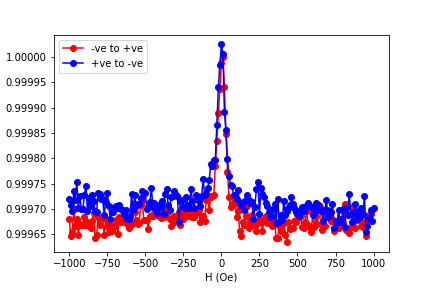
 (f)
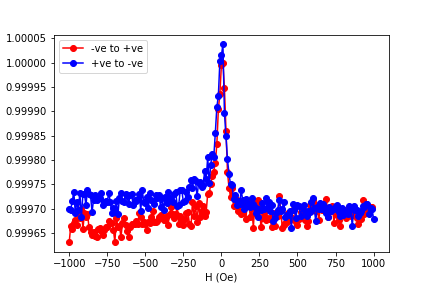


**Figure S1: GMR curves of a CFAS/Ag/CFAS GMR device with the diameter of 150 nm × 100 nm.** | Normalised GMR curves measured under an applied field of ±1 kOe (a) before and after the current crystallisation by an applied current of 500 µA for 1 s (b) once, (c) 3, (d) 10, (e) 20 and (f) 45 times. Red and blue curves measured by a sensing current of 50 µA correspond to the field sweep from negative to positive and *vice versa*.
